# Developing a hippocampal neural prosthetic to facilitate human memory encoding and recall of stimulus features and categories

**DOI:** 10.3389/fncom.2024.1263311

**Published:** 2024-02-08

**Authors:** Brent M. Roeder, Xiwei She, Alexander S. Dakos, Bryan Moore, Robert T. Wicks, Mark R. Witcher, Daniel E. Couture, Adrian W. Laxton, Heidi Munger Clary, Gautam Popli, Charles Liu, Brian Lee, Christianne Heck, George Nune, Hui Gong, Susan Shaw, Vasilis Z. Marmarelis, Theodore W. Berger, Sam A. Deadwyler, Dong Song, Robert E. Hampson

**Affiliations:** ^1^Wake Forest Baptist Medical Center, Winston-Salem, NC, United States; ^2^Department of Biomedical Engineering, University of Southern California, Los Angeles, CA, United States; ^3^Johns Hopkins Children's Center, Baltimore, MD, United States; ^4^Virginia Tech Carilion School of Medicine and Research Institute, Roanoke, VA, United States; ^5^USC Keck Memorial Hospital, Los Angeles, CA, United States; ^6^Rancho Los Amigos National Rehabilitation Hospital, Los Angeles, CA, United States

**Keywords:** neurophysiology, CA1, CA3, cognition, prosthetic, nonlinear

## Abstract

**Objective:**

Here, we demonstrate the first successful use of static neural stimulation patterns for specific information content. These static patterns were derived by a model that was applied to a subject’s own hippocampal spatiotemporal neural codes for memory.

**Approach:**

We constructed a new model of processes by which the hippocampus encodes specific memory items via spatiotemporal firing of neural ensembles that underlie the successful encoding of targeted content into short-term memory. A memory decoding model (MDM) of hippocampal CA3 and CA1 neural firing was computed which derives a stimulation pattern for CA1 and CA3 neurons to be applied during the encoding (sample) phase of a delayed match-to-sample (DMS) human short-term memory task.

**Main results:**

MDM electrical stimulation delivered to the CA1 and CA3 locations in the hippocampus during the sample phase of DMS trials facilitated memory of images from the DMS task during a delayed recognition (DR) task that also included control images that were not from the DMS task. Across all subjects, the stimulated trials exhibited significant changes in performance in 22.4% of patient and category combinations. Changes in performance were a combination of both increased memory performance and decreased memory performance, with increases in performance occurring at almost 2 to 1 relative to decreases in performance. Across patients with impaired memory that received bilateral stimulation, significant changes in over 37.9% of patient and category combinations was seen with the changes in memory performance show a ratio of increased to decreased performance of over 4 to 1. Modification of memory performance was dependent on whether memory function was intact or impaired, and if stimulation was applied bilaterally or unilaterally, with nearly all increase in performance seen in subjects with impaired memory receiving bilateral stimulation.

**Significance:**

These results demonstrate that memory encoding in patients with impaired memory function can be facilitated for specific memory content, which offers a stimulation method for a future implantable neural prosthetic to improve human memory.

## 1 Introduction

Alzheimer’s Disease (AD) and dementia is a major public health issue with over 50 million patients worldwide, resulting in spending of nearly one trillion dollars (US) per year. In the U.S. alone, an estimated 5 million cases of dementia in persons 65 years of age, or older, cost more than $250 billion per year. To date, there are no cures, and very few treatments to slow the cognitive decline associated with Alzheimer’s Disease, Parkinson’s Disease, epilepsy, stroke, head injury, and other neurological disorders (CDC, 2018).

While memory impairment is one of the most distressing symptoms of Alzheimer’s disease and age related dementia (Skaper et al., 2017), memories have different levels of importance, whether due to how they are valued by a person or urgency of the information contained within that memory. The memory of a child’s name is going to be one of great personal value, while the memory of whether the stove was left on is one of greater urgency. Content specific memory facilitation offers the future possibility of selective stimulation for memories that are especially important due to value or urgency.

When looking to restore or enhance memory function, two techniques have been shown to be effective: (1) fixed frequency stimulation designed to alter brain state (Lin et al., 2017; Kucewicz et al., 2018) and (2) spatio-temporal patterned designed to emulate ensemble activity associated with memory (Hampson et al., 2018). Fixed frequency stimulation is designed to emulate neural oscillator frequencies and influence default-mode and task-mode neural networks into a state conducive to successful memory. This stimulation paradigm has been applied to entorhinal cortex (Suthana et al., 2012; Mankin and Fried, 2020), lateral inferotemporal cortex (Jacobs et al., 2016; Ezzyat et al., 2018), and fornix (Laxton et al., 2010) in human subjects. Spatio-temporal stimulation of hippocampus has been developed (Berger et al., 2011; Hampson et al., 2013, 2018), independent of the content of memory, to focus on general brain-state aspects of memory function.

One of the greatest benefits of fixed frequency stimulation approaches is that while the set of frequencies used can be potentially customized for individuals to maximize the effect of the stimulation, it the stimulation is not based on an individual’s neural activity. Therefore, fixed frequency stimulation plans can be created that are able to be used by potentially all patients. While fixed frequency stimulation provides an approach that is able to be applied generally to patients, theories regarding the mechanism by which DBS affects neuronal networks underlying memory are varied, and even contradictory (Tan et al., 2020)—ranging from enhancing brain states associated with successful memory function (Hanslmayr and Roux, 2017), to enhancing theta (Chang et al., 2019) or gamma (Kucewicz et al., 2018) oscillations.

The potential for differing, or a combination, of mechanisms depending on the location and type of stimulation applied may explain why approaches using fixed frequency stimulations have met with mixed success, with some groups reporting no effect when stimulating hippocampus directly (Jacobs et al., 2016; Tan et al., 2020), while other groups that have shown success are dependent on a combination of differences of electrode placement, frequency and other stimulation parameters (Hanslmayr and Roux, 2017; Chang et al., 2019; Mankin and Fried, 2020; Mohan et al., 2020).

Our 2018 paper (Hampson et al., 2018) used closed-loop stimulation to “write” information “codes” into the hippocampus. These “codes” were generated by an algorithm that emulated hippocampal ensemble firing (Song et al., 2018) and was intended to reinforce hippocampal activity associated with memory. Furthermore, the model did not rely on or address specific memory content. The network properties of the nonlinear function in this method suggested the possibility of the approach for this paper, where we derived static codes for specific memory content using a Memory Decoding Model (MDM), then stimulated with these codes to enhance memory performance.

In this study we show that fixed pattern multi-site spatiotemporal codes are able to modify memory performance in a content specific manner. While we show that this stimulation approach is able to modify memory, we had a low accuracy for the derived codes. An increase in the accuracy of the derived codes is required to obtain consistent increased performance of memory. The ability to derive codes must therefore be enhanced, and doing so will likely be mostly due to an improvement in construction of training sets that are used to obtain the neural recordings that the codes are derived from. As codes are intended to be for specific information content, improved training sets that are tailored to have better association with that information content for an individual will provide better data for the model to generate stimulation patterns from. This stimulation approach, after further enhancement of the ability to derive codes, offers an alternate, and possibly complimentary, method of stimulation for use in a hippocampal prosthetic than that previously demonstrated.

## 2 Materials and methods

### 2.1 Subjects

Fourteen adult subjects were enrolled in the study (Table 1). Subjects were selected from patients with medically-refractory focal epilepsy and were undergoing seizure monitoring and localization through the use of implanted intracranial depth operative assessment, including long-term, non-invasive video-EEG analysis, pre-operative MRI, and neuropsychological assessment. Only subjects with depth electrodes in the hippocampus were selected for participation in this study.

**Table 1:** Subject demographics.

Subject	Test site	Sex	Age	Implant sites	Seizure focus	Memory function
Wake20	WFBMC	F	31	Right anterior + posterior	Extratemporal	Mild impairment
Wake21	WFBMC	F	26	Bilateral anterior	Extratemporal	Moderate impairment
Wake22	WFBMC	M	48	Bilateral anterior	Right hippocampus	Mild impairment
Wake23	WFBMC	F	51	Right anterior	Right temporal	Intact memory
Wake24	WFBMC	F	33	Left anterior	Left temporal	Intact memory
Wake28	WFBMC	F	55	Bilateral anterior	Left hippocampus	Moderate impairment
Wake29	WFBMC	F	38	Left anterior	Left temporal	Intact memory
Wake34	WFBMC	F	40	Bilateral anterior	Right hippocampus	Intact memory
Keck07	KHUSC	F	26	Bilateral anterior + posterior	Left hippocampus	Intact memory
Keck08	KHUSC	M	26	Bilateral anterior	Left hippocampus	Mild impairment
Keck12	KHUSC	M	28	Bilateral anterior + posterior	Bilateral temporal	Intact memory
Keck15	KHUSC	F	20	Bilateral anterior	Bilateral hippocampus	Mild impairment
Rancho01	RLANRH	M	35	Bilateral anterior + posterior	Bilateral hippocampus	Mild impairment
Rancho07	RLANRH	M	35	Bilateral anterior + posterior	Left hippocampus	Intact memory

All subjects were epilepsy patients undergoing Phase II intracranial monitoring at Wake Forest Baptist Medical Center (WFBMC), Keck Hospital of the University of Southern California (KHUSC) or Rancho Los Amigos National Rehabilitation Hospital (with neurosurgical services from KHUSC).

Subjects underwent all surgical procedures, post-operative monitoring, and neurocognitive testing at one of the three sites participating in this study; Wake Forest Baptist Medical Center, Keck Hospital of USC, and Rancho Los Amigo National Rehabilitation Center. This study is part of the DARPA Restoring Active Memory (RAM) project. All procedures were reviewed and approved by each locations Institutional Review Board in accordance with the National Institutes of Health. Subjects provided voluntary written informed consent prior to participation in this study that was separate from their consent for surgery.

### 2.2 Visual delayed match to sample (DMS) task with categorizable stimuli

**Figure 1:**
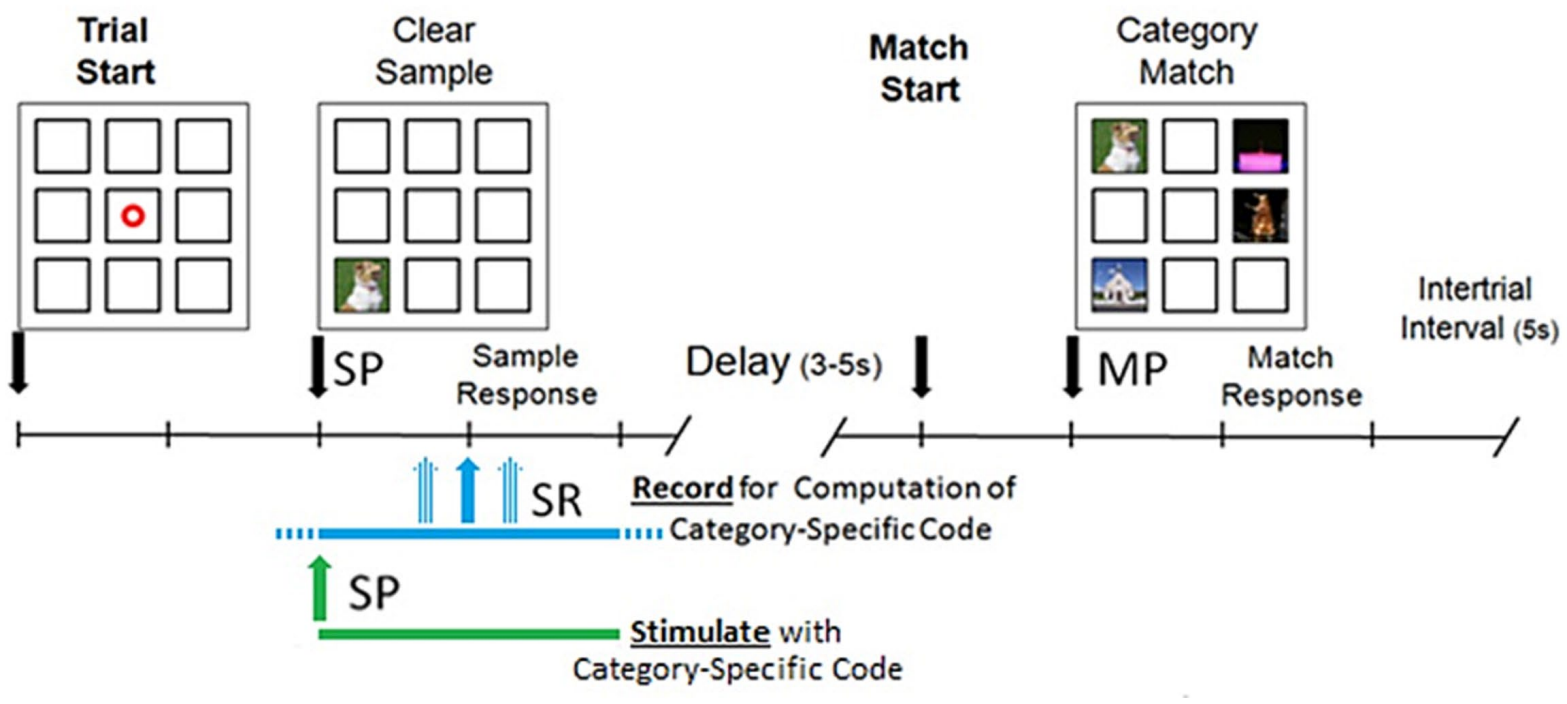
Delayed match to sample (DMS) task performed on a touch screen by patients seated in either the hospital bed or a chair. Trial is started by patient touching a focus ring which causes the sample image to be presented (SP). After patient responds by touching the sample image there is a delay and then the match image is presented (MP). Patient touches one of the images in the match phase to end the trial. During recording sessions, a 4 s window centered on SR is recorded and used for computation of Category-Specific code. During stimulation sessions, a 4 s stimulation is started at SP.

DMS sessions were performed with subjects seated in either the hospital bed or a chair with a touch screen facing them positioned for easy reach that displayed the DMS task window. The DMS task window contained nine squares in a 3 by 3 grid where images were presented for subject interaction as show in in Figure 1. The center square was only used to display a “trial start” ring that subjects touched to begin a trial. Once a trial was started, the ring disappeared and a sample image was displayed in one of the eight peripheral squares. Subjects were instructed to touch the sample image once they felt they were able to remember the image. When the sample image is touched, the squares are cleared for a delay of 3–5 s. After that delay, the matching image (i.e., identical to the sample image) and three nonmatching images were displayed in the peripheral squares. Subjects were instructed to touch the match image once they recognized it. Touching any image in the match phase ended the trial and the images were cleared from the squares. If the subject did not respond within 20 s during either the sample or match phase, the trial was ended. Once a trial was completed or timed out, there was an intertrial interval of 5 s before the trial start ring was displayed to allow the start of the next trial.

DMS sessions were run using standardized scripts of 150 trials with the sample image, match image, nonmatch images, and trial order predefined. Sample images were drawn from five categories: animal, building, plant, tool, and vehicle. One nonmatch image was drawn from one of the other four categories; one image was drawn from a group of uncategorized images that do not fit the five categories. The remaining (third) nonmatch image was drawn from a category not previously represented or from the uncategorized images. Images were not repeated within a testing day. For the first testing session of a given day, the first 20 trials contained five trials in which the nonmatch image was drawn from the same category as the match image to encourage the subject to attend to the image rather than the category. During the task, event markers (e.g., trial start, sample response, match response) were output and combined with the continuous neural recording.

### 2.3 Surgery

#### 2.3.1 Medical necessity

All subjects recruited for the study were selected from a population of patients undergoing “Phase II Intracranial Monitoring” for epilepsy diagnosis and treatment. This procedure required implantation of intracranial depth electrodes for localization of seizure origin and spread. Clinical considerations included epilepsy refractory to multiple anti-epileptic drugs and/or other treatments, and noninvasive monitoring long term video-electroencephalography (vEEG), pre-operative magnetic resonance imaging (MRI), magnetoencephalography (MEG) if available, and neuropsychological assessment. Depth electrode placement was determined by pre-surgical EEG seizure monitoring.

Subjects underwent all surgical procedures, post-operative monitoring, and neurocognitive testing at one of the three sites participating in this study; Wake Forest Baptist Medical Center, Keck Hospital of USC, and Rancho Los Amigo National Rehabilitation Center. All procedures were reviewed and approved by each locations Institutional Review Board: IRB00023148 (WFBMC), HS-16-00068 (KHUSC), IRB#: 221 (RLANRH), and in accordance with the National Institutes of Health and Department of the Navy Human Research Protection Oversight. Subjects provided voluntary written informed consent specifically for participation in this study, separate from their consent for surgery, medical, and Phase II procedures.

#### 2.3.2 Electrode implantation and removal

Implantation and removal of electrodes was performed in the same manner as reported in Hampson et al. (2018). Epilepsy patients typically received 8–10 “Macro” style EEG probes, and 1–4 “Macro–Micro” style EEG/single neuron probes in a single surgical procedure. Due to changes in electrode availability, probes were sourced from both Ad-Tech Medical Instrumentation Corporation (Racine, WI) and PMT Corporation (Chanhassen, MN) for this study. Post-operative localization of electrode placements were verified by a combination of MRI (Figure 1) and electrophysiological activity consistent with putative hippocampal CA1 and CA3 principal cells [including nonlinear MIMO model (Song et al., 2016b) and pairwise cross-correlations (Wicks et al., 2020)]. Electrodes in all subjects were explanted after seizure localization was confirmed or at a time designated by each subject’s care team after a sufficient period of invasive monitoring had been performed.

### 2.4 Hippocampal neural recording

Neuronal recordings were performed in the same manner as reported previously (Hampson et al., 2018). Subjects had between one to four “macro–micro” depth electrodes placed in the hippocampus. Single neuron extracellular action potential waveforms were isolated and identified for online and offline sorting of single unit discharges. Continuous electrical digitized monitoring identified single unit action potential waveforms (bandpass filtered to 500–5,000 Hz, 30,000 samples/s), and single unit spike events (i.e., timestamps, 200 μs resolution) during DMS task performance. Recorded data included both neural spike activity and task event markers (e.g., trial start, sample response, match response).

Recording sessions were performed on the second day post-implantation to allow the subject to fully recover from the effects of anesthesia. Each recording session consisted of DMS training for 150 trials. A minimum of 100 successful trials were desired for use in model generation to allow for at least 20 trials for each image category. Subjects were monitored during the recording session to ensure that they remained alert and responsive throughout the session. Recordings and associated behavioral control scripts were sent to Dr. Dong Song’s staff for processing and model computation.

### 2.5 Stimulation model and paradigm

#### 2.5.1 Memory decoding model (MDM)

The MDM is a content-specific derivation of the MIMO model reported previously (Song et al., 2016a, 2017). The MIMO model was a nonlinear input–output model that continuously predicted CA1 “output” from CA3 “input,” and did not rely or use information content of DMS images. In contrast, the MDM correlated MIMO-based CA1 neural firing predictions with the trial image category (Song et al., 2016a; Geng et al., 2018; Sandler et al., 2018; Yu et al., 2018; She et al., 2021) and produced a sparse static stimulation pattern for each category for each patient. The MDM model therefore allowed the creation of these static stimulation patterns outside of a stimulation session, while the MIMO model would be run during a session to generate trial specific stimulation patterns live. Briefly, B-spline basis functions are used to extract memory features from spatio-temporal patterns of spikes (Song et al., 2013, 2014). B-splines are piecewise polynomial functions with smooth transitions between adjacent pieces at a set of interior knot points, where the number of knots determines the number of B-splines.

Images presented in the sample phase are labelled by normal human subjects with 29 non-mutually exclusive categories and features (Song et al., 2016a; She et al., 2022), to a target signal for the classification model and simplify it to a binary variable. The classification model is thus based on logistic regression. For a given B-spline knot sequence in each 10-fold cross-validation/estimation trial, one set of are estimated and used to reconstruct the sparse classification function matrix (SCFM) which is used to calculate the conditional probability of the modeled label with a given input spatiotemporal pattern.

The performance of each static stimulation pattern generated by the model is evaluated with the Matthews correlation coefficients (MCCs). MCC is robust to unbalanced data and can be calculated from the confusion matrix of true positives, true negatives, false positives, and false negatives. The MCC value varies between −1 and 1, with MCCs of 1, 0 and −1 represent perfect prediction, no better than random prediction, and completely opposite prediction, respectively. The averaged SCFM across all temporal resolutions is thus calculated as the MCC-weighted summation of individual SCFMs, representing the spatio-temporal characteristics of the classification model estimated with all trials and resolutions.

Only out-of-sample MCCs are utilized for the SCFMSs and only SCFMs are considered for the MDM. Full details of the model are provided by Song et al. (2013, 2014).

**Figure 2:**
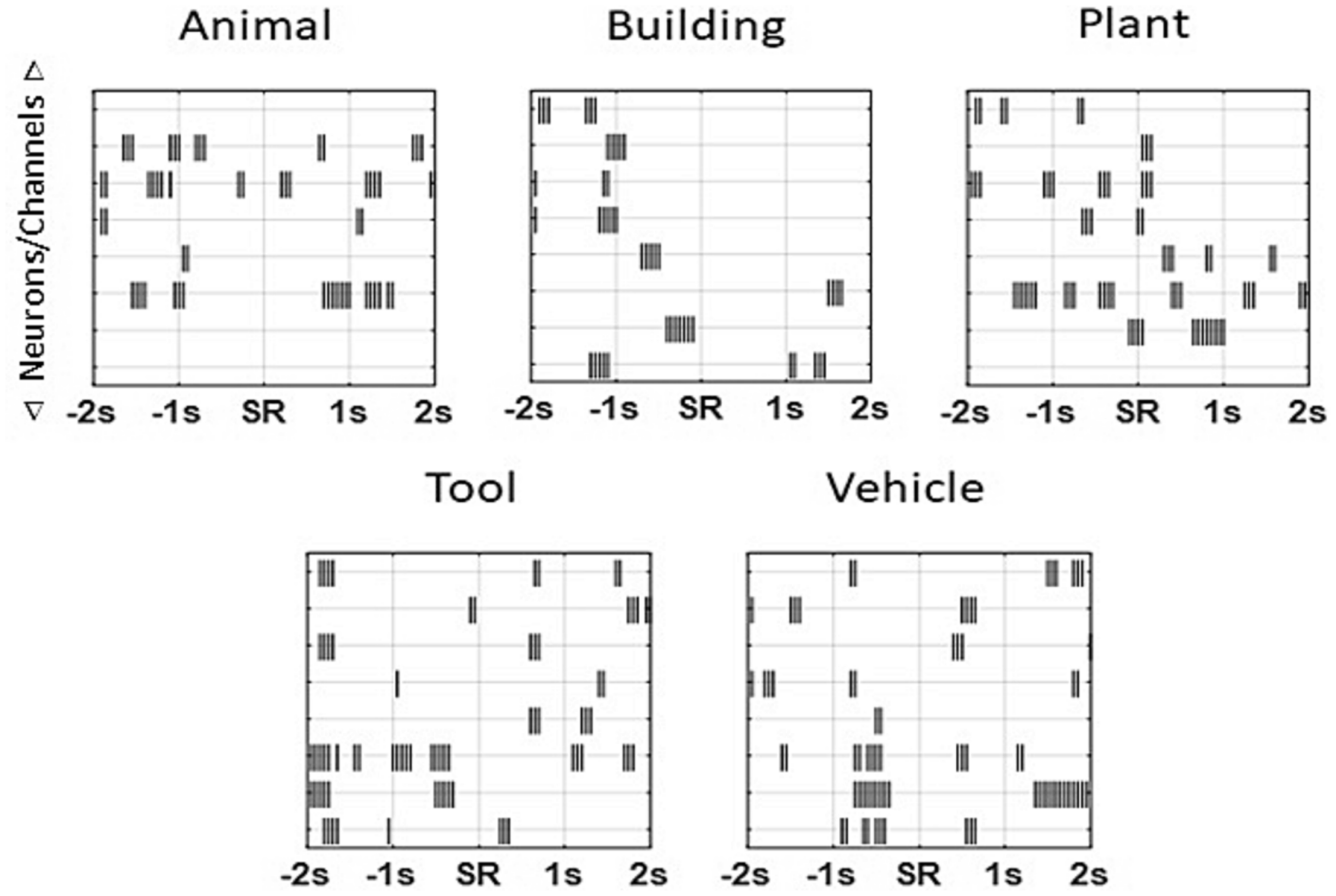
Sample patterns from an individual patient for each of the categories. Unique patterns that were 4 s in length were derived for each subject for every patient using the MDM applied to each patient’s own neural recordings. Each “tick” marks a single stimulation pulse (1 ms duration, 150 μA constant current ~1 V, biphasic, 50 ms per phase) on a channel. As multiple neurons could be isolated on an individual trial during a recording session, the MDM model calculated stimulation patterns by channel and not by neuron. Subsequent stimulation pulses for a given channel occurred no sooner than 50 ms (20 Hz). Note theta-frequency-like clusters (~4 Hz) in some categories.

Stimulation patterns for each patient were created for the five image categories used in the study; animal, building, plant, tool, and vehicle. Neural activity in a 4 s window centered on the sample response for each trial during a patient’s recording session was used to calculate the MDM model for that patient. Stimulation patterns were generated using a two-step model: (1) calculation of a pattern that was derived from the recorded activity for all trials that were within a category, (2) elimination of elements of the pattern that were common across categories. The MDM approach created fixed patterns corresponding to a patient’s neural activity associated with and unique to each of the categories. As the generated patterns were derived to be specific for the category content, they were sparse codes with a lower density firing pattern than patterns generated by the MIMO model. Following computation of the MDM model (Figure 2) Matlab (Mathworks, Natick, MA) scripts containing the patterns were supplied to the appropriate clinical testing team for assessment on the same subject from whom the MDM was computed.

#### 2.5.2 Neuronal stimulation

The MDM stimulation was supplied through the use of Matlab array scripts compatible with a custom Matlab program operating the Blackrock Cerestim R96 microstimulator. The program delivered stimulation through the same CA1 microelectrode sites via which neural activity was recording on the first (recording) session. A given category pattern was selected for a given trial under control of a trial-script that specified sample/match and nonmatch images for each DMS trial to ensure adequate distribution of trial types across a session. While the MIMO-model calculated the stimulation pattern in real-time based on neural activity, the MDM-derived stimulation pattern was a fixed stimulation pattern. Therefore, while the MIMO model required re-isolation of the neurons so that the spike sorting on the stimulation day was as similar as possible to that on the recording day, this was not necessary for use of the MDM-derived stimulation. Nevertheless, re-isolation of neurons to a close match of the prior session was performed to allow for live monitoring of neural and stimulation activity during a stimulation session.

Random stimulation was applied on a limited number of trials for Wake20, Wake21, Wake22, Wake23, and Wake 24, in the same manner as was used when testing the MIMO model as in Hampson et al. (2018). This stimulation was used only to validate that the random stimulation was having the same affect in this patient group as had been seen in the patient group for the Hampson et al. (2018) paper. Once the results were validated there was deemed no reason to continue retesting random stimulation in all patients.

### 2.6 Patient testing

#### 2.6.1 DMS stimulation session

Memory testing using MDM model category-specific patterns consisted of a DMS session, during which stimulation was applied, and an associated Delayed Recognition (DR) session during which memory retention was tested. The delay between the Sample and Match phase during a DMS trial is of insufficient time to test memory using the clipart images that were used in this experiment. The DMS task therefore is not able to be used to test memory. The DMS task is used to present the clipart images to the patient and ensure that the patient gives attention to the images, as the patient must pay attention to the images to complete the task. Typical error frequency in the DMS task is two errors or fewer in a 150 trial session, both in DMS recording and stimulation sessions. DMS stimulation sessions were nearly identical to DMS recording sessions, except for the spatio-temporal patterned electrical stimulation applied on select trials. Stimulation commenced with the presentation of the sample image, for a duration of 4 s. A maximum of 16 independent (CA1) channels could be stimulated per subject, each stimulation pulse was 1 ms duration, 150 μA constant current ~1 V, biphasic, 50 ms per phase. Subsequent stimulation pulses for a given channel occurred no sooner than 50 ms (20 Hz). During DMS sessions the average time sample image presentation and sample response by the subject was ~1.9 s, resulting in near simultaneity of stimulation with the memory encoding interval in Figure 1.

Trials within the stimulation session consisted of non-stimulated trials (NoStim), Match stimulated trials, non-target stimulated trials (NonMatch), and Random stimulated trials (RandomStim). NoStim trials were used as within subject controls. Match stimulated trials were trials where the pattern used to stimulate the subject was the same category as the sample image, for example an animal category stimulation was paired with presentation of an animal sample image. These trials were used to test the effectiveness of stimulation to enhance memory. NonMatch stimulated trials were trials where the pattern used to stimulate was from one of the four categories that did not match the category of the sample image, e.g., a building category stimulation paired with a plant sample image. These trials were used as a control for non-specific properties effects of stimulation that did not depend on the image-specific information, e.g., the electrical or timing properties of the stimulus. RandomStim trials were used to compare to RandomStim trials in Hampson et al. (2018).

#### 2.6.2 Delayed recognition (DR) memory testing

**Figure 3:**
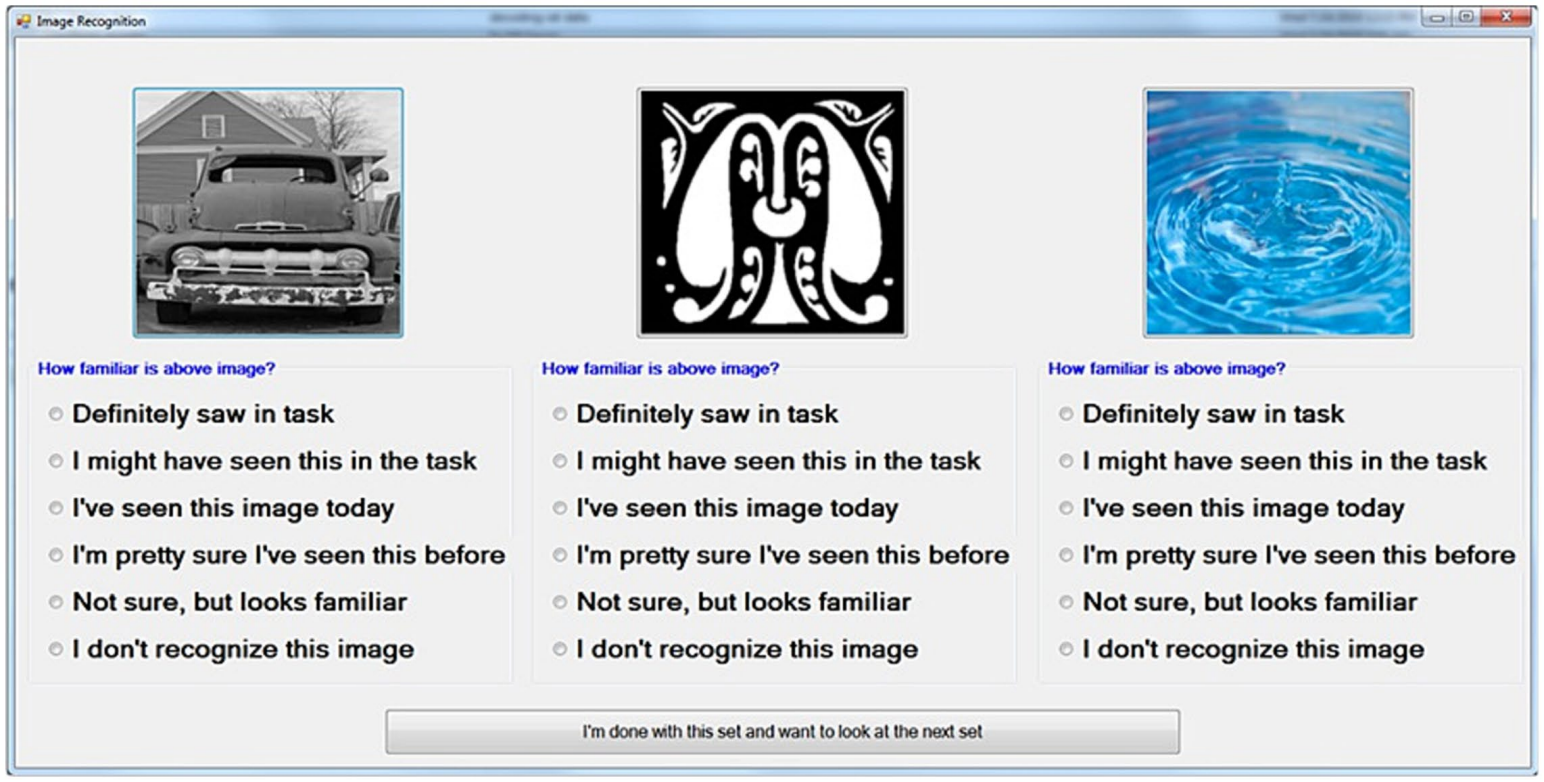
Diagram of an individual-trial DR screen following DMS stimulation session. Images consist of the sample/match image, one Nonmatch image from the same trial, and one novel image not used in any prior testing. The “familiarity” choices displayed with each image are converted to a numeric ranking (0 = Do not recognize, 5 = Definitely saw) for scoring and analysis.

DR sessions commenced 15–20 min after a DMS stimulation session to produce a range of memory retention from approximately 15–75 min. The order of trials in the DR session was shuffled randomly from the order in the DMS stimulation session to allow for the variations in that time span and to prevent memorization of sequences. DR trials consisted of three images presented at a time (Figure 3), with the Sample/Match image, one Nonmatch image, and one Novel image from a given DMS trial. Subjects rated each image with respect to familiarity. The Novel image did not appear in any DMS trial, and was not repeated in any of the test sessions on a given day. Left-to-right order was also shuffled between trials to avoid presenting the Sample, Nonmatch and Novel images in consistent locations. Subjects were instructed on the rating system at the start of the DR session. The DR task window included short text next to each rating option to remind the subject of the verbal instructions. Subjects were observed and assisted by testing staff through the DR session to ensure that they did not have difficulty understanding and applying the ratings.

Each rating was subsequently converted to a numerical value for outcome scoring. The highest rating of familiarity (“Definitely saw in Task,” Figure 3) was scored as 5, while the least familiarity (“Not sure, but looks familiar”) was scored as 1. If a subject did not rate an image, or specified that an image had no familiarity, the image was scored as 0.

DR trial outcome was then determined by three conditions. To be considered correct, the Sample image had to be scored greater than 2, the Sample must be ranked greater than or equal to both the Nonmatch image rank and the Novel image rank. Any trial in which the Sample ranking was less than 3, or was ranked less than either the Nonmatch image or the Novel image, was scored as an error response.

### 2.7 Statistical analysis

#### 2.7.1 T-test statistical analysis of DMS task performance

A two tailed t-test was used to determine if stimulation had an influence on individual patient performance within the DMS task. The t-test was used for within-subject analyses given large numbers of individual DMS trials. The null hypothesis was that there would be no difference between nonstimulated and stimulated trial performance. This hypothesis was due to the ease of the DMS task due to the short time between the Sample and Match phases. Note that the results of this test were also used to set mean, variance, and probability expectations for further analysis.

For each patient, DMS trials were first sorted by the category type of the Sample image; animal, building, plant, tool, vehicle, and uncategorized. This allowed for examination as to whether a patient performed differently on trials with specific image types. Patient performance on DMS trials for every image category was compared to the DMS performance for every other image category. Using a P threshold of 0.025, the null hypothesis was upheld, with no significant difference in DMS performance between trials in any image category for any patient.

DMS trials were then sorted by the type of stimulation received during the DMS trial; Match Stim, NonMatch Stim, NoStim, and RandomStim. Performance for each stim type was compared to performance of all other stim types. This allowed for examination as to whether stimulation modified patient performance compared to non stimulated trials, and if so whether it was for all stimulation types or only specific stimulation types. Using a P threshold of 0.025, the null hypothesis was upheld, with no significant difference in DMS performance between trials for any stimulation type in any patient.

#### 2.7.2 χ^2^ statistical analysis of DR task performance

The χ^2^ test was used to compare performance on the DR task between trials that received Match Stim and NoStim, as well as between trials that received NonMatch Stim and NoStim, with a null hypothesis that Stim vs. NoStim did not alter the probability with which a correct DR trial occurred. χ^2^ values were calculated for overall performance for each patient, performance within each category for each patient. In two patients, Wake20 and Rancho01, analysis was not able to be made for all categories as there were not sufficient trials in all categories to be able to allow a statistical analysis to be performed. Note that patient subjects could be additionally sorted by memory impairment status as well as whether they had received bilateral or unilateral stimulation; therefore, overall performance both for individual categories and all categories was computed for these subgroups.

For stimulated trials, when calculating the χ^2^ value, the expected values were calculated from the observed values for the stimulated and non stimulated trials. The expected value for non-stimulated trials was calculated based on the population data for non-stimulated trials.

**Figure 4:**
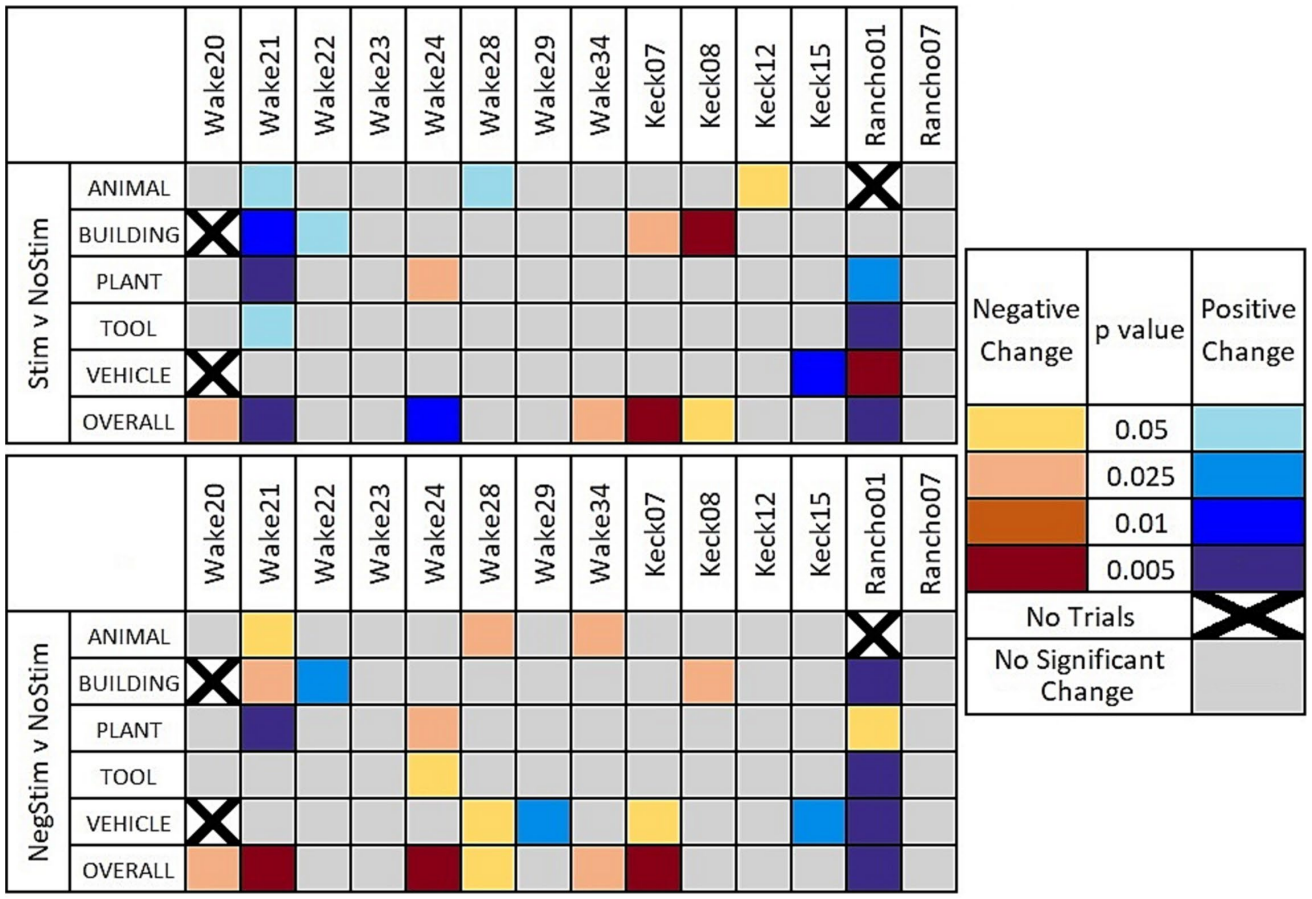
Heat maps of significant results. Significant results shown between Match Stim vs. NoStim (top row) and NonMatch Stim vs. NoStim (bottom row) per subject and category. Significant results with a decrease in performance are indicated with yellow and orange, and increased performance with blue. Grey indicates no significant difference in performance. Squares with black Xs indicate combinations with too few trials to compute.

**Figure 5:**
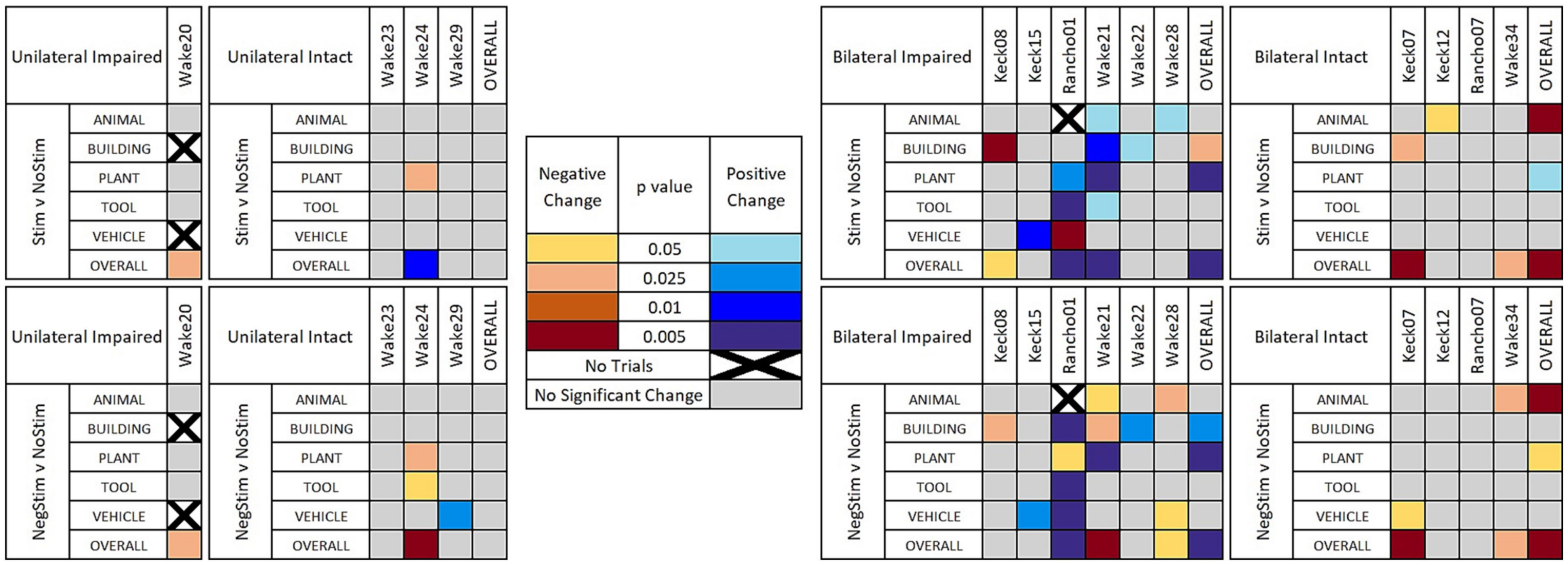
Heat maps of significant results. Significant results shown between Match Stim vs. NoStim (top rows) and NonMatch Stim vs. NoStim (bottom rows) per subject and category, broken down by unilateral or bilateral stimulation, and by impaired or intact memory in subjects. Significant results with a decrease in performance are indicated with yellow and orange, and increased performance with blue. Grey indicates no significant difference in performance. Squares with black Xs indicate combinations with too few trials to compute.

Figure 4 shows a heat map that indicates when the χ^2^ value was determined to reach a critical value for different levels of statistical significance between MatchStim and NoStim, as well as NonMatchStim and NoStim trials. The heat map also shows whether the change in performance due to stimulation was an increase in performance, or a decrease in performance. Figure 5 shows heat maps for the patients broken down by memory impairment status and laterality of stimulation received.

RandomStim trials for all patients tested with this trial type (Wake20-Wake24), showed a statistically significant lower performance on RandomStim trials compared to NoStim trials.

#### 2.7.3 Calculation of statistical power

The average number of trials completed by a patient was 111.79 trials, with the lowest number of trials being 80 and the highest 134. The average number of Match Stim trials per patient ranged from 6.46 for animal trials to 6.92 for building trials. The average number of NonMatch Stim trials per patient ranged from 5.50 for tool trials to 5.92 for animal and vehicle trials. The average number of NoStim trials per patient ranged from 10.29 for tool trials to 11.29 for plant trials. This resulted in a statistical power of approximately 10% for each combination of image category and stimulation type within patient. While this is lower than the ideal 80%, power in a chi square is frequently not the same as the power in a t-test or ANOVA due to the asymmetric (i.e., nongaussian) distribution of the chi square.

With respect to the low overall statistical power, achieving high power would require an extremely large number of trials, which would take over an order of magnitude more time with a patient than what is possible in a clinical setting due to the time allotted for experiments. Within the clinical restrictions, and without causing undue fatigue on the patients, this experiment has run the maximum number of trials that was able to be performed with each patient. This is a restriction that is inherent with working with not only a patient population, but also the low number of overall trials compliant with a memory task (e.g., Suthana et al., 2012; Ezzyat et al., 2018) required to perform these types of experiments. Further, due to the relatively small nature of the patient population undergoing procedures which require the implantation of neural electrodes, this study, with an n of 14, has a relatively large patient population compared to many published neurostimulation papers. The low statistical power and limited patient group is therefore not unique to our lab or experiment, but also applies to other research that has been published in this area.

## 3 Results

### 3.1 DMS-DR scoring

The delay between the Sample and Match phase during a DMS task trial is of insufficient time to test memory using the clipart images that were used in this experiment. Performance on the DMS task was examined to determine whether individual trials needed to be excluded based on DMS performance. Two trials, out of over 1,500, across all subjects had timeouts that occurred and these two trials were excluded from the data.

As there was no difference within patient between groups of trial when sorted either by stim type, or by Sample image category, no further DMS trials were excluded from DR analysis.

**Figure 6:**
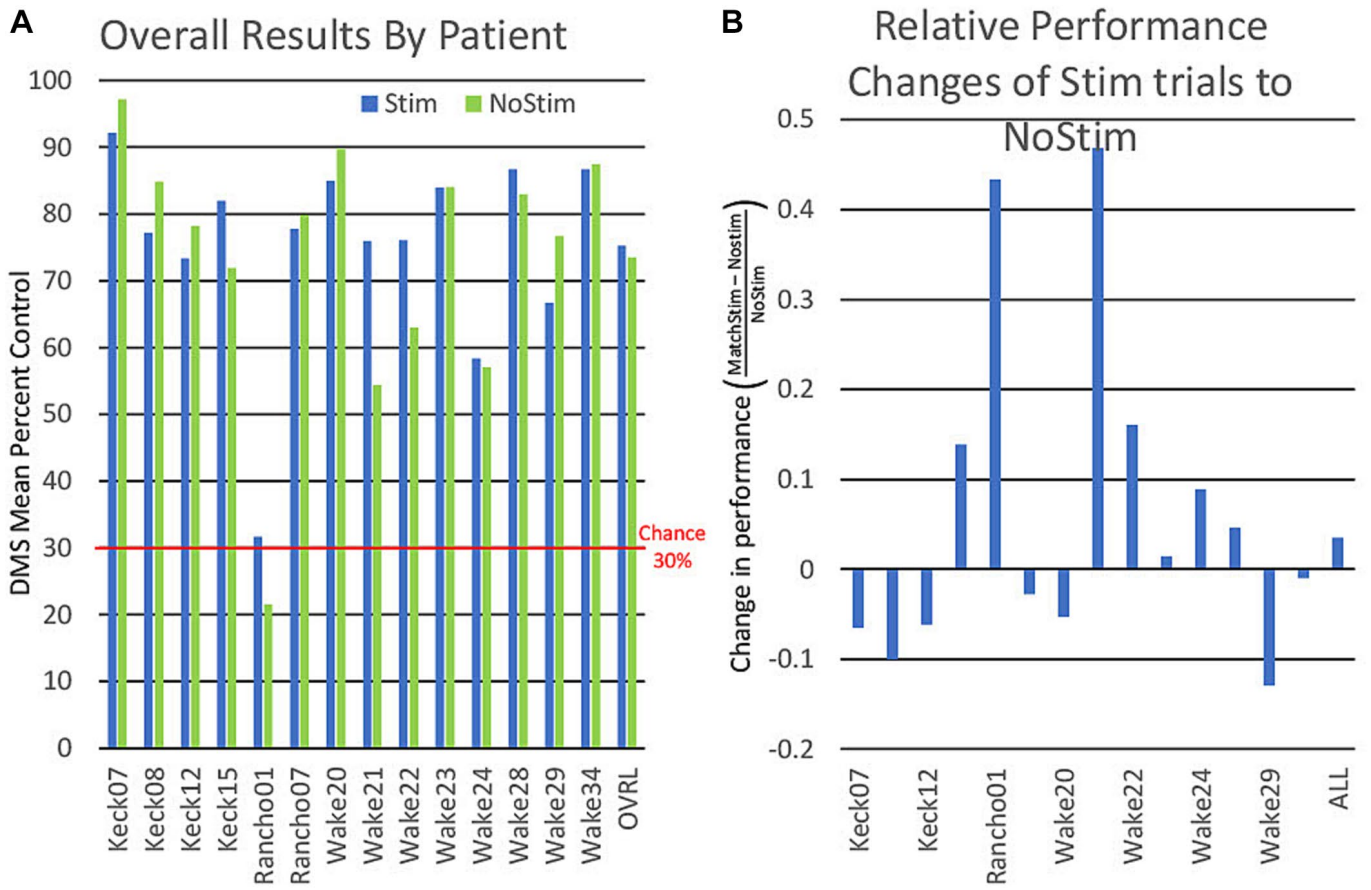
**(A)** Graph of individual patient performance on stimulated and non stimulated trials. DMS performance is for all trials combined and is not an average of performance within categories. **(B)** Graph showing the relative differences of patient performance between stimulated and non stimulated trials using non stimulated trials as a baseline.

For the DR analysis, a comparison of MatchStim trials to NoStim trials by subject, showed approximately one-half of subjects increased performance on stimulation trials while the remainder showed decreased performance (Figure 6). Across all subjects, there was a small increase in performance on Match Stim trials over NoStim trials. The overall performance which combined all categories, may suggest that MDM stimulation was only effective on some subjects. Though comparison of Match Stim to NoStim shows a slight increase in DMS-DR performance, it combines the performance of the five categories of MDM stim (animal, building, plant, tool, and vehicle) and does not take into account category differences within a subject.

**Figure 7:**
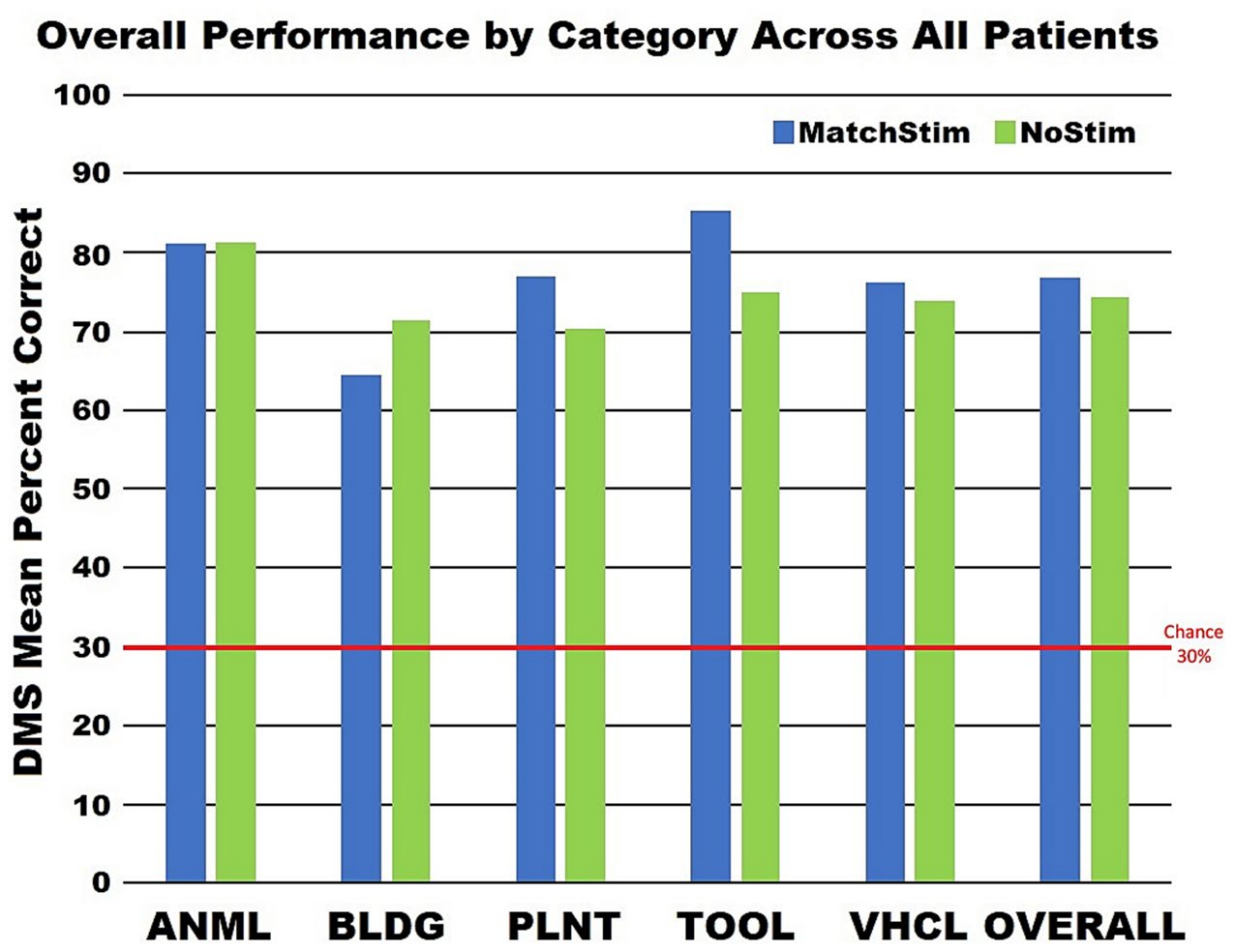
Graph of category performance on stimulated and non stimulated trials. Performance for each category was calculated by combining all trials from all patients and not by averaging the performances of the patients within a category.

Sorting DMS-DR results by category (Figure 7) yields a finer distinction, with performance improvements in three categories (plant, tool, vehicle) where Match Stim performance exceeded NoStim performance. In contrast, the animal category produced similar performance between Match Stim and NoStim, while the Match Stim for the building category was decreased relative to NoStim. This differential result due to subject-x-category sorting suggests that effectiveness of MDM stim should be evaluated not just on a subject or category basis alone, but by examining the results for each category for each subject. That the stimulations patterns within a category are not the same patterns, but are unique patterns for each patient which are generated from each patient’s own neural recordings further reinforces that effectiveness of the MDM stimulation should be examined based on results for each category for each patient.

Sorting DMS-DR results by subject and category (Figure 4) shows variation in and across both subjects and categories, reinforcing the need to consider two potential ramifications of MDM-based stimulation: (1) whether the MDM predicts effective stimulation patterns, and (2) whether the trial image within a category were sufficiently homogeneous to enable the MDM to extract a coherent category of information. Answering these two questions required an additional scoring of the DMS-DR performance to facilitate comparisons across subjects and categories.

### 3.2 Effectiveness of stimulation by subject and category

#### 3.2.1 Random stim vs. no stim

RandomStim was used in only five patients, Wake20-Wake24. RandomStim had previously been used as a control during our testing of closed loop stimulation that reinforced patient’s native hippocampal activity that we reported in the Hampson et al. paper in 2018. In Hampson et al. (2018) RandomStim did not increase patient performance on the task, but resulted in similar or lower performance than on NoStim trials (Hampson et al., 2018). Results of RandomStim in Wake20-Wake24 were consistent with the RandomStim in Hampson et al. (2018), therefore continued testing of RandomStim was discontinued in the patients following these.

#### 3.2.2 Match stim vs. no stim

Figure 4 reveals that overall performance for a subjects does not necessarily indicate how a given subject responded on individual categories. For example, when comparing Match Stim to No Stim for Wake24 (Figure 4, top panel) it can be seen the subject had a significant decrease in the plant category, but when all trials were grouped together showed a significant overall increase in performance due to Match Stim. Wake 20 and Wake 34 showed no significant results in individual categories, but showed an overall decrease in performance. In contrast, Keck07, Keck08, Rancho01, and Wake21 showed overall results that correspond well to the significant results in individual categories.

A comparison of MDM effects on Match Stim relative to NoStim [9 of 67 combinations of subject and category (cells in Figure 4, top)] showed a statistically significant increase in performance, while 5 combinations showed a statistically significant decrease in performance. Thus, 13.4% of the time MDM stimulation resulted in significantly facilitated performance relative to NoStim. Furthermore, when considering whether MDM stimulation affected DMS-DR performance in any direction, 20.9% of combinations of subject and category showed significant positive or negative effects of the MDM Match Stim. However, when looking at overall performance by patients only 3 showed overall increases in performance while 4 showed an overall decrease.

Breaking down results by whether stimulation was Unilateral or Bilateral, and whether the patient had Intact or Impaired memory according to presurgical neurocognitive testing, as shown in Figure 5 (top row), gives a more nuanced and clearer picture of when MDM stimulation may be beneficial. In 4 patients with unilateral stimulation, only Wake 24 showed a significant change in category performance, which was a decrease in Plant trials, while still showing an overall increase in performance. In the 4 patients that received Bilateral stimulation and had Intact memory, no patient showed an increase in any category on MatchStim trials. In contrast, 5 out of 6 patients with Impaired memory that received Bilateral stimulation showed a significant increase in performance in at least one category, with Wake 21 showing an increase in 4 categories, and Rancho01 showing an increase in 2 and a decrease in 1.

#### 3.2.3 Nonmatch stim vs. no stim

For NonMatch stim (Figure 4, bottom), 7 of 67 combinations showed a statistically significant increase in performance relative to NoStim, while 9 showed a statistically significant decrease. It was expected that NonMatch stim, representing a mismatch between the MDM prediction and the composition of the category, should produce an overall decrease in DMS-DR performance. Thus, it is unsurprising that more category combinations show a decrease (13.4%) than an increase (10.4%), and that the number of decreases are greater than that in Match Stim relative to NoStim.

While the number of subjects with overall significant changes of performance remains the same as with Match Stim relative to NoStim, the proportion of those alterations that are a decrease in performance is greater. Additionally, the animal category shows an overall significant decrease in performance across all patients. Along with the number of significant decreases being greater overall with NonMatch Stim, there were two subjects that had more than one category that showed a significant decrease in results. Three out of the 7 increases in performance were in Rancho01 who is of note as having the lowest performance of all patients across all stim types, with their NoStim performance being less than half of the next lowest NoStim performance. This is nearly half of the significant increases in performance due to NonMatch stim, but Rancho01 had less than a quarter of the significant increases due to Match Stim. This, combined with the decrease in performance shown due to RandomStim, suggests that while any stimulation does not have an enhancing effect, the stimulation patterns may not be as specific to their titular categories. Therefore, there may be an overlap between what MDM patterns are nominally stimulating, and what they are actually stimulating for. If such is the case, then Rancho01 perhaps was more easily influenced by overlapping stimulation due to their low baseline performance.

Further sorting results by Unilateral or Bilateral stimulation, and memory status, as in Figure 5 (bottom row) offers evidence that the stimulation patterns derived by the MDM model are not as specific to a category as intended. The 4 patients with Unilateral stimulation and Intact memories showed a decrease in performance in two category and patient combinations and an increase in one, compared to the single decrease due to MatchStim. Patients with Bilateral stimulation and intact memory showed similar performance between MatchStim and NonMatch stim. In contrast, patients with Bilateral stimulation and Impaired memory still show the most positive results, as they did with MatchStim, but the ratio of increased to decreased performance changed from a ratio of 9:2 to a ratio of 1:1.

## 4 Discussion

Category-based, content-specific code stimulation significantly alters memory delayed recognition in 22.4% of instances and 37.9% of instances in which the patient had impaired memory and received bilateral stimulation, clearly indicating that MDM-based stimulation has the potential to be used to significantly modify memory.

That all but one case of performance enhancement of a category for a patient was in patients that received Bilateral stimulation and had Impaired memory suggests that MDM based stimulation may be useful as a model to control a neural prosthetic to restore memory function, but only with certain prerequisites. Differing types of stimulation approaches for patients with different conditions is consistent with the results shown in Roeder et al. (2022).

The mix between positive and negative influence in patients that received Bilateral stimulation and with Impaired memory is indicative that MDM categories are not as specific to the nominal category as desired. This may be reduced by improvement in the composition of images that the MDM model used to create category specific stimulation patterns. The differential positive and negative effects suggest that MDM-derived predictions of CA1 ensemble firing are not necessarily specific to the intended information content of five image categories used here. This is consistent with stimulation patterns being generated by the MDM model not for individual images, but for a collection of images grouped by category.

Given that these images are composed of natural content (flowers, grass, sky, etc.) with the potential for brightly colored foreground and/or background objects, it is possible that much of this category overlap is due to color content of images. Nevertheless, the result that 22.4% of subject and category combinations memory was influenced by the MDM-based stimulation, and that 39.7% of subject and category combinations memory for patients that received Bilateral stimulation and have Impaired memory, supports the conclusion that, when applied under the appropriate conditions, stimulation of hippocampus with fixed spatio-temporal patterns intended to mimic ensemble representation of ensemble encoding of mnemonic information can alter human memory retention.

There are multiple challenges involved in deriving a static code for specific information content in a visual memory task. The first is whether the subjects will associate the images with the memory content that the researchers intend for the image to be associated with. Location of content within an image has been shown to contribute to the focus and potential importance of a portion of an image to a person (Kiat et al., 2022). An image of a house with a tree in it could be intended to have the house be the main focus of the image by researchers, but the position of the tree could interfere with this intent. Salience of image components can also be affected by age, as salience of image components is affected by knowledge gained over time (Rehrig et al., 2023), which can result in differing focus on image components between individuals that is influence by age. Interest in components of images can also vary between subjects. An image of a penguin on an iceberg might have the penguin be of greater interest to one subject, while the iceberg and the natural scene it is part of be of greater interest to another subject.

The second challenge is the level of abstractness of the memory content that codes are attempted to be created for. In this study five categories were chosen; Animal, Building, Plant, Tool, and Vehicle. These categories are a higher level of abstract in comparison to more simple content compared to content like basic colors such as yellow, blue, green, and red. While it would have been preferable to attempt to test this stimulation approach using content codes derived for less abstract information, the goal to enhance more higher level abstract information was a core component of the DARPA RAM (Restoring Active Memory) program which funded this research.

In comparison with other demonstrations of memory-enhancing stimulation in humans, our previous report showed 34–37% improvement in memory overall as a result of stimulating hippocampal CA1 ensembles with a pattern derived from the continuous nonlinear MIMO model (Hampson et al., 2018). Ezzyat et al. (2017) showed that temporal lobe DBS stimulation produced 12–25% improvement in free recall of word lists. While up to 100% improvement was seen in some subjects when a closed-loop implementation was used, it should be noted that the quantity of remembered items was limited (approx. 12 words per list) as was the duration of retention (up to 10 min.) (Ezzyat et al., 2018). In contrast, Jacobs et al. (2016) showed 20–50% impairment in memory with fixed-frequency DBS stimulation of hippocampus, and Merkow et al. (2017) emphasized the role of stimulation to enhance forgetting, rather than memory retention. For further examination of the rich diversity in effects of DBS-like stimulation for memory facilitation (see Mankin and Fried, 2020). Note that the nonlinear model (Hampson et al., 2018) at the root of the stimulation technique used here provided some of the best memory-facilitation among the papers cited.

The success of fixed-frequency DBS techniques for other neurological conditions urges further investigation of adapted for use with dementia, despite—or because of—the studies cited above (Suthana and Fried, 2014). Studies which show that successful memory function is represented by brain states measurable in terms of theta (Lin et al., 2017) and beta-gamma (Kragel et al., 2017) frequencies and robust theta-gamma coupling (Jones et al., 2020; Radiske et al., 2020), strongly supports the need for research into theta range stimulation of the hippocampus as well as an examination of whether MIMO and MDM stimulation utilized here had similar effects on theta oscillations in hippocampus.

Development of neural prosthetics to restore and facilitate memory can use different approaches. The results of this study indicate that fixed pattern hippocampal stimulation is a viable approach for altering retention of specific information content in human subjects. MDM stimulation altered memory in nearly a quarter of the instances, with a nearly 2 to 1 ratio in increase to decrease across all patients when Match Stim was used, and a 9 to 2 ratio in Impaired memory patients that received Bilateral stimulation. These results indicate that MDM model stimulation can be of benefit to patients with impaired memory, but refining of training for the MDM model and accuracy of derived codes is needed prior to this approach being able to be ready for use in a neural prosthetic.

Future directions for this work will focus on three issues raised by the results of this study: (1) the need for further examination of the effectiveness of NonMatch Stim, (2) the influence of non category specific image features that can be shared across categories and (3) the possibility that MDM stimulation codes derived from one (or more patients) can be applied successfully to another patient. Thus we intend to examine neural firing for other categories, such as color or background features of the DMS-DR images, determine the influence of common features such as color, colors on the derived patterns for different categories, and test whether it is possible to “transfer” stimulation patterns between subjects (see Deadwyler et al., 2013). An open question is also whether MDM stimulation is writing a memory code, or reinforcing codes that are already present. Each of these questions will move the research forward to the point of developing a memory prosthetic operating on general features of memory encoding that are common across patients, yet specific enough to facilitate retention of specific memory content.

## Data availability statement

The raw data supporting the conclusions of this article will be made available by the authors, without undue reservation.

## Ethics statement

The studies involving humans were approved by Wake Forest Baptist Medical Center Institutional Review Board, Keck Hospital of USC Institutional Review Board, and Rancho Los Amigos National Rehabilitation Center Institutional Review Board. The studies were conducted in accordance with the local legislation and institutional requirements. Written informed consent for participation in this study was provided by the participants’ legal guardians/next of kin. Written informed consent was obtained from the individual(s) for the publication of any potentially identifiable images or data included in this article.

## Author contributions

BR: Conceptualization, Data curation, Formal analysis, Investigation, Methodology, Software, Supervision, Validation, Visualization, Writing – original draft, Writing – review & editing. XS: Formal analysis, Investigation, Methodology, Software, Writing – review & editing. AD: Software, Writing – review & editing. BM: Formal analysis, Investigation, Methodology, Software, Writing – review & editing. RW: Investigation, Writing – review & editing. MW: Conceptualization, Investigation, Writing – review & editing. DC: Investigation, Writing – review & editing. AL: Investigation, Writing – review & editing. HC: Investigation, Validation, Writing – review & editing. GP: Investigation, Writing – review & editing. CL: Investigation, Writing – review & editing. BL: Investigation, Writing – review & editing. CH: Investigation, Writing – review & editing. GN: Investigation, Writing – review & editing. HG: Investigation, Writing – review & editing. SS: Investigation, Writing – review & editing. VM: Conceptualization, Methodology, Visualization¸ Writing – review & editing. TB: Conceptualization, Funding acquisition, Methodology, Visualization¸ Writing – review & editing. SD: Conceptualization, Funding acquisition, Project administration, Supervision, Writing – review & editing. DS: Conceptualization, Investigation, Methodology, Project administration, Software, Supervision, Visualization, Writing – review & editing. RH: Conceptualization, Data curation, Formal analysis, Funding acquisition, Investigation, Methodology, Project administration, Resources, Software, Supervision, Validation, Visualization, Writing – original draft.

## References

[ref1] Berger T. W. Hampson R. E. Song D. Goonawardena A. Marmarelis V. Z. Deadwyler S. A. (2011). A cortical neural prosthesis for restoring and enhancing memory. J. Neural Eng. 8:046017. doi: 10.1088/1741-2560/8/4/046017, PMID: 21677369 PMC3141091

[ref2] CDC. (2018). At a glance: Alzheimer's disease. Available at: https://www.cdc.gov/aging/publications/aag/alzheimers.html

[ref3] Chang C. W. Lo Y. C. Lin S. H. Yang S. H. Lin H. C. Lin T. C.. (2019). Modulation of theta-band local field potential oscillations across brain networks with central thalamic deep brain stimulation to enhance spatial working memory. Front Neurosci 13:1269. doi: 10.3389/fnins.2019.01269, PMID: 32038122 PMC6988804

[ref4] Deadwyler S. A. Berger T. W. Sweatt A. J. Song D. Chan R. H. Opris I.. (2013). Donor/recipient enhancement of memory in rat hippocampus. Front Syst. Neurosci 7:120. doi: 10.3389/fnsys.2013.00120, PMID: 24421759 PMC3872745

[ref5] Ezzyat Y. Kragel J. E. Burke J. F. Levy D. F. Lyalenko A. Wanda P.. (2017). Direct brain stimulation modulates encoding states and memory performance in humans. Curr. Biol 27, 1251–1258. doi: 10.1016/j.cub.2017.03.028, PMID: 28434860 PMC8506915

[ref6] Ezzyat Y. Wanda P. A. Levy D. F. Kadel A. Aka A. Pedisich I.. (2018). Closed-loop stimulation of temporal cortex rescues functional networks and improves memory. Nat. Commun. 9:365. doi: 10.1038/s41467-017-02753-0 29410414 PMC5802791

[ref7] Geng K. Shin D. C. Song D. Hampson R. E. Deadwyler S. A. Berger T. W.. (2018). Mechanism-based and input-output modeling of the key neuronal connections and signal transformations in the CA3-CA1 regions of the hippocampus. Neural Comput. 30, 149–183. doi: 10.1162/neco_a_01031, PMID: 29064783

[ref8] Hampson R. E. Song D. Opris I. Santos L. M. Shin D. C. Gerhardt G. A.. (2013). Facilitation of memory encoding in primate hippocampus by a neuroprosthesis that promotes task-specific neural firing. J. Neural Eng. 10:066013. doi: 10.1088/1741-2560/10/6/066013, PMID: 24216292 PMC3919468

[ref9] Hampson R. E. Song D. Robinson B. S. Fetterhoff D. Dakos A. S. Roeder B. M.. (2018). Developing a hippocampal neural prosthetic to facilitate human memory encoding and recall. J. Neural Eng. 15:036014-032552/aaaed036017. doi: 10.1088/1741-2552/aaaed7, PMID: 29589592 PMC6576290

[ref10] Hanslmayr S. Roux F. (2017). Human memory: brain-state-dependent effects of stimulation. Curr. Biol. 27, R385–R387. doi: 10.1016/j.cub.2017.03.079, PMID: 28535389

[ref11] Jacobs J. Miller J. Lee S. A. Coffey T. Watrous A. J. Sperling M. R.. (2016). Direct electrical stimulation of the human entorhinal region and hippocampus impairs memory. Neuron 92, 983–990. doi: 10.1016/j.neuron.2016.10.062, PMID: 27930911

[ref12] Jones K. T. Johnson E. L. Berryhill M. E. (2020). Frontoparietal theta-gamma interactions track working memory enhancement with training and tDCS. Neuroimage 211:116615. doi: 10.1016/j.neuroimage.2020.116615, PMID: 32044440 PMC7733399

[ref13] Kiat J. E. Hayes T. R. Henderson J. M. Luck S. J. (2022). Rapid extraction of the spatial distribution of physical saliency and semantic informativeness from natural scenes in the human brain. J. Neurosci. 42, 97–108. doi: 10.1523/JNEUROSCI.0602-21.2021, PMID: 34750229 PMC8741164

[ref14] Kragel J. E. Ezzyat Y. Sperling M. R. Gorniak R. Worrell G. A. Berry B. M.. (2017). Similar patterns of neural activity predict memory function during encoding and retrieval. Neuroimage 155, 60–71. doi: 10.1016/j.neuroimage.2017.03.042, PMID: 28377210 PMC5789770

[ref15] Kucewicz M. T. Berry B. M. Kremen V. Miller L. R. Khadjevand F. Ezzyat Y.. (2018). Electrical stimulation modulates high gamma activity and human memory performance. eNeuro 5, ENEURO.0369–ENEU17.2018. doi: 10.1523/ENEURO.0369-17.2018, PMID: 29404403 PMC5797477

[ref16] Laxton A. W. Tang-Wai D. F. McAndrews M. P. Zumsteg D. Wennberg R. Keren R.. (2010). A phase I trial of deep brain stimulation of memory circuits in Alzheimer's disease. Ann. Neurol. 68, 521–534. doi: 10.1002/ana.22089, PMID: 20687206

[ref17] Lin J. J. Rugg M. D. Das S. Stein J. Rizzuto D. S. Kahana M. J.. (2017). Theta band power increases in the posterior hippocampus predict successful episodic memory encoding in humans. Hippocampus 27, 1040–1053. doi: 10.1002/hipo.22751, PMID: 28608960 PMC6517838

[ref18] Mankin E. A. Fried I. (2020). Modulation of human memory by deep brain stimulation of the entorhinal-hippocampal circuitry. Neuron 106, 218–235. doi: 10.1016/j.neuron.2020.02.024, PMID: 32325058 PMC7347298

[ref19] Merkow M. B. Burke J. F. Ramayya A. G. Sharan A. D. Sperling M. R. Kahana M. J. (2017). Stimulation of the human medial temporal lobe between learning and recall selectively enhances forgetting. Brain Stimul. 10, 645–650. doi: 10.1016/j.brs.2016.12.011, PMID: 28073638 PMC5410394

[ref20] Mohan U. R. Watrous A. J. Miller J. F. Lega B. C. Sperling M. R. Worrell G. A.. (2020). The effects of direct brain stimulation in humans depend on frequency, amplitude, and white-matter proximity. Brain Stimul. 13, 1183–1195. doi: 10.1016/j.brs.2020.05.009, PMID: 32446925 PMC7494653

[ref21] Radiske A. Gonzalez M. C. Conde-Ocazionez S. Rossato J. I. Kohler C. A. Cammarota M. (2020). Cross-frequency phase-amplitude coupling between hippocampal theta and gamma oscillations during recall destabilizes memory and renders it susceptible to reconsolidation disruption. J Neurosci 40, 6398–6408. doi: 10.1523/JNEUROSCI.0259-20.2020, PMID: 32661022 PMC7424867

[ref22] Rehrig G. Hayes T. R. Henderson J. M. Ferreira F. (2023). Visual attention during seeing for speaking in healthy aging. Psychol. Aging 38, 49–66. doi: 10.1037/pag0000718, PMID: 36395016 PMC10021028

[ref23] Roeder B. M. Riley M. R. She X. Dakos A. S. Robinson B. S. Moore B. J.. (2022). Patterned hippocampal stimulation facilitates memory in patients with a history of head impact and/or brain injury. Front. Human Neurosci. 16:933401. doi: 10.3389/fnhum.2022.933401, PMID: 35959242 PMC9358788

[ref24] Sandler R. A. Geng K. Song D. Hampson R. E. Witcher M. R. Deadwyler S. A.. (2018). Designing patient-specific optimal neurostimulation patterns for seizure suppression. Neural Comput. 30, 1180–1208. doi: 10.1162/neco_a_01075, PMID: 29566356

[ref25] She X. Berger T. W. Song D. (2021). A double-layer multi-resolution classification model for decoding spatio-temporal patterns of spikes. Neural Comput. 34, 219–254. doi: 10.1162/neco_a_01459, PMID: 34758485 PMC9470026

[ref26] She X. Robinson B. S. Flynn G. Berger T. W. Song D. (2022). Accelerating input-output model estimations with parallel computing for testing hippocampal memory prostheses in human. J. Neurosci. Methods 370:109492. doi: 10.1016/j.jneumeth.2022.109492, PMID: 35104492

[ref27] Skaper S. D. Facci L. Zusso M. Giusti P. (2017). Synaptic plasticity, dementia and Alzheimer disease. CNS Neurol. Disord. Drug Targets 16, 220–233. doi: 10.2174/1871527316666170113120853, PMID: 28088900

[ref28] Song D. Hampson R. E. Robinson B. S. Marmarelis V. Z. Deadwyler S. A. Berger T. W. (2016a). Decoding memory features from hippocampal spiking activities using sparse classification models. Conf. Proc. IEEE Eng. Med. Biol. Soc. 2016, 1620–1623. doi: 10.1109/EMBC.2016.7591023, PMID: 28268639

[ref29] Song D. Harway M. Marmarelis V. Z. Hampson R. E. Deadwyler S. A. Berger T. W. (2014). Extraction and restoration of hippocampal spatial memories with non-linear dynamical modeling. Front Syst. Neurosci 8:97. doi: 10.3389/fnsys.2014.00097, PMID: 24904318 PMC4036140

[ref30] Song D. Robinson B. Hampson R. Marmarelis V. Deadwyler S. Berger T. (2016b). Sparse large-scale nonlinear dynamical modeling of human hippocampus for memory prostheses. IEEE Trans. Neural Syst. Rehabil. Eng 10, 272–280. 10.1109/TNSRE.2016.2604423 PMC5623111 28113595

[ref31] Song D. Robinson B. S. Hampson R. E. Marmarelis V. Z. Deadwyler S. A. Berger T. W. (2018). Sparse large-scale nonlinear dynamical modeling of human hippocampus for memory prostheses. IEEE Trans. Neural Syst. Rehabil. Eng. 26, 272–280. doi: 10.1109/TNSRE.2016.2604423, PMID: 28113595 PMC5623111

[ref32] Song D. She X. Deadwyler S. A. Berger T. W. (2017). Multi-resolution multi-trial sparse classification model for decoding visual memories from hippocampal spikes in human. Conf. Proc. IEEE Eng. Med. Biol. Soc. 2017, 1046–1049. doi: 10.1109/EMBC.2017.8037006 29060053

[ref33] Song D. Wang H. Tu C. Y. Marmarelis V. Z. Hampson R. E. Deadwyler S. A.. (2013). Identification of sparse neural functional connectivity using penalized likelihood estimation and basis functions. J. Comput. Neurosci. 35, 335–357. doi: 10.1007/s10827-013-0455-7, PMID: 23674048 PMC3805829

[ref34] Suthana N. Fried I. (2014). Deep brain stimulation for enhancement of learning and memory. Neuroimage 85, 996–1002. doi: 10.1016/j.neuroimage.2013.07.066, PMID: 23921099 PMC4445933

[ref35] Suthana N. Haneef Z. Stern J. Mukamel R. Behnke E. Knowlton B.. (2012). Memory enhancement and deep-brain stimulation of the entorhinal area. N. Engl. J. Med 366, 502–510. doi: 10.1056/NEJMoa1107212, PMID: 22316444 PMC3447081

[ref36] Tan S. Z. K. Fung M. L. Koh J. Chan Y. S. Lim L. W. (2020). The paradoxical effect of deep brain stimulation on memory. Aging Dis. 11, 179–190. doi: 10.14336/AD.2019.0511, PMID: 32010491 PMC6961776

[ref37] Wicks R. T. Witcher M. R. Couture D. E. Laxton A. W. Popli G. Whitlow C. T.. (2020). Hippocampal CA1 and CA3 neural recording in the human brain: validation of depth electrode placement through high-resolution imaging and electrophysiology. Neurosurg Focus 49:E5. doi: 10.3171/2020.4.FOCUS20164, PMID: 32610296

[ref38] Yu G. J. Bouteiller J. C. Song D. Berger T. W. (2018). Decoding position to analyze spatial information encoding in a large-scale neuronal network model of rat dentate gyrus. Annu. Int. Conf. IEEE Eng. Med. Biol. Soc. 2018, 6137–6140. doi: 10.1109/EMBC.2018.8513576, PMID: 30441735 PMC6391309

